# C-Reactive Protein Kinetic as a Potential Predictive and Prognostic Factor during Treatment with Checkpoint Inhibitors in R/M-HNSCC

**DOI:** 10.3390/cancers16132424

**Published:** 2024-06-30

**Authors:** Frederic Jungbauer, Claudia Scherl, Nicole Rotter, Annette Affolter, Anne Lammert, Elena Seiz, Margot Thiaucourt, Lena Huber

**Affiliations:** 1 Department of Otorhinolaryngology, Head- and Neck-Surgery, University Medical Centre Mannheim, Medical Faculty Mannheim of Heidelberg University, 68167 Mannheim, Germany; 2 MVZ Labor Dr. Limbach & Kollegen, 69126 Heidelberg, Germany

**Keywords:** head and neck squamous cell carcinoma (HNSCC), prognostic factor, checkpoint inhibitors (CPI), PD-1, C-reactive protein (CRP), flare dynamic

## Abstract

**Simple Summary:**

This study investigates the prognostic value of C-reactive protein (CRP) kinetics in patients with recurrent or metastatic squamous cell carcinoma of the head and neck (R/M-HNSCC) treated with checkpoint inhibitors (CPI). We analyzed data from 44 patients treated between 2018 and 2023, applying two existing CRP classifications and developing a new one. While the existing classifications did not correlate with overall survival (OS), our novel CRP kinetic classification showed a significant association with OS (*p* = 0.05). Multivariate analysis identified our CRP classification (*p* = 0.007) and the outcome of the first re-staging (*p* = 0.002) as independent prognostic factors. These findings suggest that our new CRP kinetic classification could serve as a valuable prognostic marker for R/M-HNSCC patients undergoing CPI therapy, though further validation with larger patient cohorts is needed.

**Abstract:**

Introduction The kinetic of C-reactive protein (CRP) in the early phase of therapy with checkpoint inhibitors (CPI) and its prognostic value has already been investigated in several tumor entities. In particular, flare dynamics have been described as a positive prognostic parameter. The aim of this retrospective study is to examine the extent to which such an application can also be transferred to patients with recurrent or metastatic squamous cell carcinoma of the head and neck region (R/M-HNSCC). Material and Methods All patients treated with CPI for R/M-HNSCC at our clinic between 2018 and 2023 were included (*n* = 44). Demographic, clinical, histopathologic and laboratory data were extracted from the digital patient records and statistically analyzed. We then examined the CRP kinetic using two previously published classifications and proposed a new classification ourselves. Subsequently, correlation analyses were performed with the overall survival (OS) of the patients. Results Of the two CRP kinetic classifications previously published, only one showed a correlation with the result of the first re-staging, and neither showed a correlation with the OS of R/M-HNSCC patients. Our new CRP kinetic classification showed a significant association with OS in R/M-HNSCC patients (*p* = 0.05). In a multivariate analysis, our CRP kinetic classification (*p* = 0.007) and the outcome of the first re-staging (*p* = 0.002) were significant independent factors for OS. Discussion Our novel CRP kinetic classification significantly correlates with OS in R/M-HNSCC patients, indicating a potential prognostic marker. Existing classifications from other cancer entities showed limited prognostic significance, emphasizing the need for tailored markers. For validation, however, testing on larger R/M-HNSCC patient collectives is necessary.

## 1. Introduction

Patients with head and neck squamous cell carcinoma who have non-operable recurrences or distant metastases (R/M-HNSCC) can be treated with checkpoint inhibitors (CPI) for palliative purposes. The antibodies nivolumab and pembrolizumab bind to the programmed death 1 (PD-1) protein, a cell receptor, thus blocking binding by the ligand PD-L1 and preventing immunosuppression by the tumor cells. However, the response of patients to therapy is highly variable [1]. Because only some patients benefit in terms of a response to therapy (remission or stable disease), the identification of biomarkers for early assessment of this is a critical component of individualized oncological therapy. While prognostic factors have so far mostly focused on the expression of PD-1 in the tumor, the reaction of the patient’s immune system is also becoming increasingly interesting for research. For example, the ratio of neutrophil granulocytes to lymphocytes is thought to be a prognostic marker in CPI therapy of HNSCC patients [2]. 

The C-reactive protein (CRP) is a polypeptide molecule belonging to the family of pentraxins. It is primarily produced in the liver in response to pro-inflammatory cytokines [3]. Currently, its concentration in the blood is determined in routine clinical practice primarily for the laboratory chemical diagnosis of inflammation and infections. However, it is a relatively unspecific parameter, and its dynamics are subject to a certain time latency. 

Meta-analyses have so far demonstrated a negative prognostic value for pre-therapeutically elevated CRP levels in HNSCC patients [4]. In addition, the role of preoperative CRP to lymphocyte ratio in HNSCC patients has been suggested as a possible prognostic tool [5]. With regard to immunotherapy of HNSCC patients, a prognostic value of the CRP to albumin ratio has also been postulated [6]. However, all these data were examined in relatively small patient collectives and must, therefore, be interpreted with caution. 

Previous studies in other tumor entities have shown that the dynamics of CRP at the start of CPI therapy could have a prognostic value for the outcome of patients. This has been demonstrated in patients with renal cell carcinoma [7,8], lung carcinoma [9,10] and urothelial carcinoma [11]. Such a prognostic value was also recently suggested for patients with HNSCC [12]. 

Of particular interest here is the flare dynamic. This refers to an increase in CRP at the start of CPI therapy with a subsequent drop. In the aforementioned studies, so-called responders and non-responders were also defined with regard to CRP kinetic.

In 2021, Fukuda et al. defined the three groups as follows [7]: CRP levels increased to more than double compared with baseline within one month after initiation of CPI therapy (flare) and then decreased to a lower value than baseline within three months (CRP flare-responders); CRP levels decreased by ≥30% within three months without “flare” (CRP responders). The remaining patients who met neither the first nor the second definition were declared non-responders. The subsequent studies mentioned above adopted this definition.

A further assessment of CRP kinetics in patients with renal cell carcinoma was already proposed by Ishihara et al. in 2020 [13]. Here, the patients were also divided into three groups: If the baseline CRP was below 10 mg/L, patients were labeled as “normal”. If the baseline CRP was above 10 mg/L but fell below 10 mg/L in the nadir within the first three months of CPI therapy, they were described as “normalized”. If the baseline CRP was above 10 mg/L and did not fall below 10 mg/L within three months, they were described as “non-normalized”. Here, too, the authors described a significant association of CRP dynamics with overall survival (OS), with the longest survival in the “normal” group, intermediate survival in the “normalized” group and poorer survival in the “non-normalized” group.

This retrospective study was conducted to examine the extent to which the early dynamics of CRP could also have a prognostic value in patients with HNSCC undergoing CPI therapy. 

## 2. Material and Methods

All patients treated with a PD-1 checkpoint inhibitor (nivolumab or pembrolizumab) as a monotherapy for R/M-HNSCC at our Department of Otorhinolaryngology, Head and Neck Surgery between 2018 and 2023 were included in the study (*n* = 48). The CRP measurements were carried out directly before the respective administration of CPI therapy. As a result, CRP levels were measured every 2 weeks in patients receiving nivolumab and every 3 weeks in patients receiving pembrolizumab. CRP was measured on the same day as the blood sample was taken using an immunoassay (Atellica CH C-Reactive Protein_2, Siemens Healthineers, Erlangen, Germany). Patient demographic and clinical data were extracted from the digital medical record and correlated with patient survival data along with laboratory chemistry parameters.

Survival analysis based on baseline CRP levels was conducted using both Cox regression models for absolute values and Kaplan–Meier estimation, categorizing CRP levels as normal (≤5 mg/L) or elevated (>5 mg/L).

First, the prognostic value of the two previously postulated CRP kinetic definitions was tested. We then described our own definition of the “flare” dynamic: a “flare” dynamic should be assumed if CRP1 is lower than CRP2 and CRP3 lower than CRP2, i.e., the CRP increased in the first control measurement during CPI therapy compared to the baseline measurement and then fell again in the next measurement.

Patient characteristics and oncological outcomes were compared among the three groups using the χ^2^ test for categorical variables and analysis of variance for numerical variables. Logistic regression analysis was used to identify predictive factors for an objective response. In each group, OS was estimated using the Kaplan–Meier method, and differences between the groups were assessed using the logrank test. In multivariate analyses for OS, the Cox proportional hazards regression model was used. For this analysis, the sex, the age cohort of the patients (younger or older than 70 years), the p16 status of the tumor, the result of the first re-staging and the three different CRP group models were used. OS was evaluated as the time from CPI initiation to the date of death or last follow-up. The analysis was performed with R Statistical Software (v4.1.2; R Core Team 2021). A *p*-value ≤ 0.05 was considered significant. The data is given with the interquartile range (IQR) and range.

The study was conducted according to the guidelines of the Declaration of Helsinki and approved by the local ethics committee of the University of Heidelberg (approval number 2023-891).

## 3. Results

There were *n* = 44 patients, including 33 men (75%) and 11 women (25%). The mean age was 66.3 years (IQR 13.25 years, 44–91 years). Nivolumab was given to 16 patients (36%) at 2-week intervals and pembrolizumab to 28 patients (63%) at 3-week intervals. The primary tumor is located in the oropharynx in 25 patients (56.8%), in the hypopharynx in 7 patients (15.9%), in the oral cavity in 4 patients (9%) and in the larynx in 3 patients (6%). One patient each had a carcinoma of the nasal cavity, the paranasal sinus, the parotid gland, the auditory canal and a carcinoma of unknown primary (2.2% each). The first re-staging after 12 weeks via CT scan, according to the RECIST criteria [14], showed stable disease in 7 patients (16%), progressive disease in 20 patients (45%), partial remission in 14 patients (32%) and a mixed response in 1 patient (2%). A total of 2 patients died before the first re-staging (4%). Progressive disease is further described as non-favorable, stable disease, mixed response and remission as favorable re-staging. 

**Figure 1:**
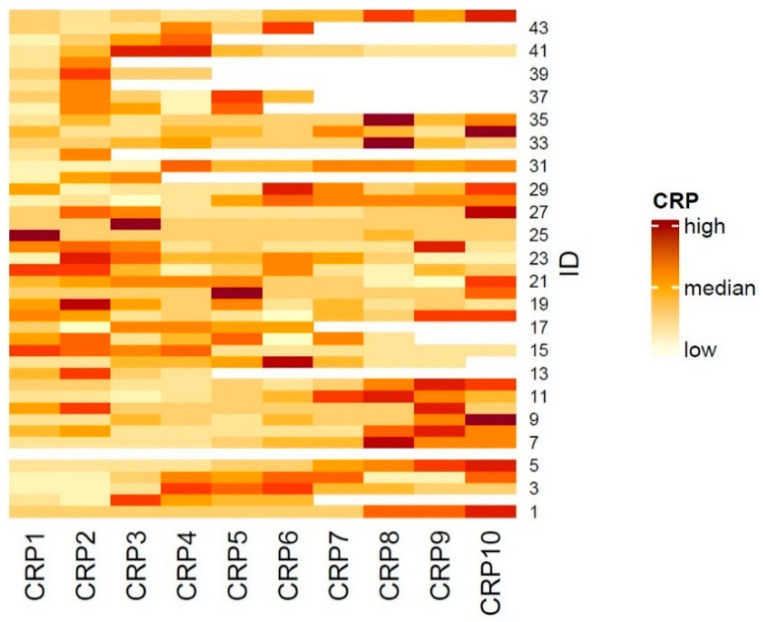
Heatmap visualizing the CRP concentration at baseline (CRP1) and over the course of CPI therapy (CRP2–CRP10, before administration of the next immunotherapy) in the different patients of the collective (Pat.-ID). White fields mark missing values for deceased patients.

The median baseline concentration at the start of CPI therapy (CRP1) is 17.75 mg/L (IQR 43.25 mg/L, 0–118 mg/L). Figure 1 shows a heat map of the dynamics of the CRP concentration over the course of the first nine CRP measurements (CRP2–CRP10), measured before each of the first 10 doses of immunotherapy.

There are no significant associations between OS and patients’ baseline CRP, nor is there a group difference between patients with a normal CRP at baseline ≤5 mg/L and patients with an elevated CRP (*p* = 0.9).

**Figure 2:**
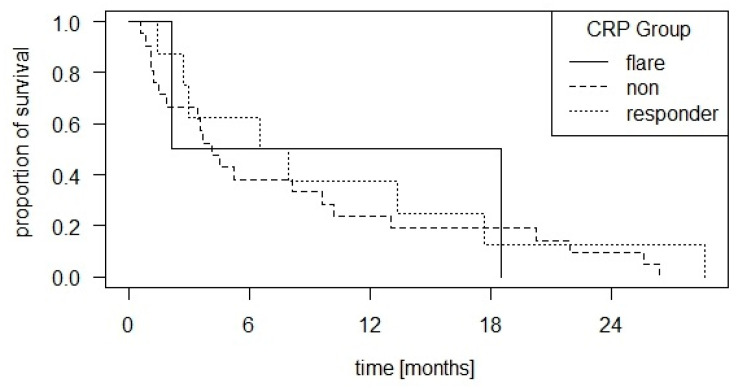
Kaplan–Meier curve of overall survival with group distribution according to CRP dynamics as defined by Fukuda et al. [7]: CRP levels increased to more than double compared with baseline within one month after initiation of CPI therapy and then decreased to a lower value than baseline within three months (CRP flare); CRP levels decreased by ≥30% within three months without “flare” (CRP responders); neither of the first two definitions (CRP non-responders). There were no significant survival differences in the logrank test (*p* = 0.7).

Based on the CRP group definitions of Fukuda et al. [7], there are 5 patients (11%) with flare dynamics, 11 patients (25%) with responder dynamics and 28 patients (64%) with non-responder dynamics. There are no significant survival differences in the logrank test (*p* = 0.7). The corresponding survival curves are shown in Figure 2. 

**Figure 3:**
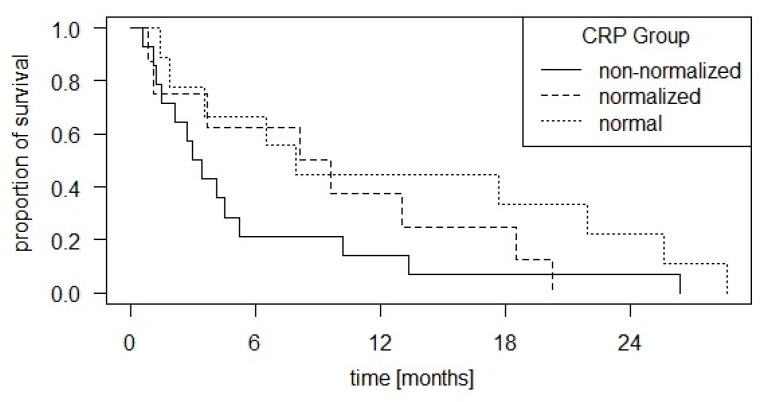
Kaplan–Meier curve of overall survival with group distribution according to CRP dynamics as defined by Ishihara et al. [13]: If the baseline CRP was below 10 mg/L, patients were labeled as “normal”. If the baseline CRP was above 10 mg/L but fell below 10 mg/L in the nadir within the first three months of CPI therapy, they were described as “normalized”. If the baseline CRP was above 10 mg/L and did not fall below 10 mg/L within three months, they were described as “non-normalized”. There were no significant survival differences in the logrank test (*p* = 0.2).

Based on the CRP group definitions of Ishihara et al. [13], there are 16 patients (36%) with “normal” dynamics, 13 patients (30%) with “normalized” dynamics and 15 patients (34%) with “non-normalized” dynamics. There are also no significant survival differences in the logrank test (*p* = 0.2). The corresponding survival curves are shown in Figure 3. The combination of “normal” and “normalized” vs. the “non-normalized” group also failed to reach the significance level (*p* = 0.1).

Our own definition of the CRP categories results in 15 patients (34%) with flare and 29 (66%) patients with non-flare dynamics. 

**Figure 4:**
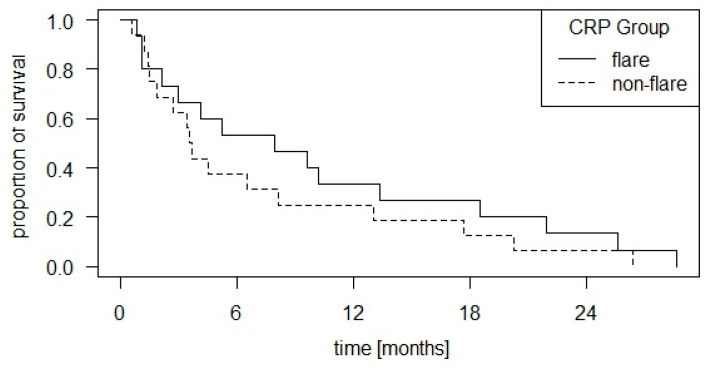
Kaplan–Meier curve of overall survival with group distribution according to CRP dynamics by own definition: If the CRP concentration increased after the first CPI administration compared to the baseline measurement and decreased again after the subsequent administration, this was assessed as a “flare”, otherwise as a “non-flare”. There was a significant difference in survival between the two CRP groups (*p* = 0.05) in favor of the “flare” group.

With this group definition, there is a significant difference in survival between the two CRP groups (*p* = 0.05) in favor of the “flare” group. The curves are shown in Figure 4.

**Figure 5:**
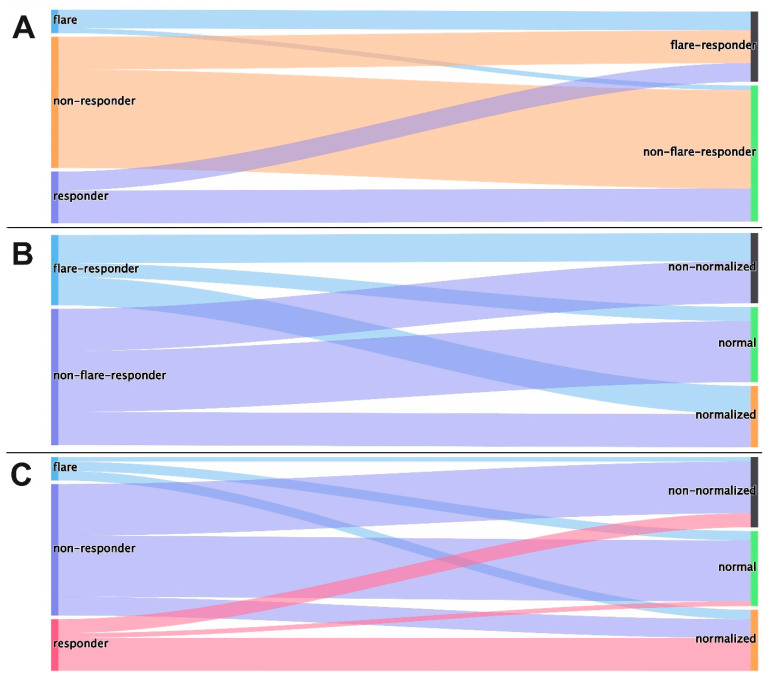
Comparison of the assignments of the different CRP dynamic groups according to the different groups as a Sankey diagram: (**A**) Fukuda et al. [7] (**left**) and own definition (**right**), (**B**) own definition (**left**) and Ishihara et al. [13] (**right**) and (**C**) Fukuda et al. [7] (**left**) and Ishihara et al. [13] (**right**).

Figure 5 shows a Sankey diagram comparing the assignments of the various CRP kinetic groups. It can be seen that the assignment to the groups that were assessed as prognostically favorable in the individual studies (“flare” and “responder” in the definition of Fukuda et al., “normal” and “normalized” in the definition of Ishihara et al. and “flare-responder” in our definition) shows some overlaps, but that there are also relevant divergences in the classification and interpretation. 

No significant group difference is observed in terms of OS in patients with p16-positive or p16-negative carcinomas. Similarly, no distinction is found between patients with and without toxic use (nicotine, alcohol or both). Additionally, OS did not significantly differ with regard to the TNM stage or the localization of the primary tumor. There is also no significant difference with regard to gender, the medication used (pembrolizumab or nivolumab) and the age of the patients at the start of CPI therapy. There are no significant associations between PD-L1 levels or the neutrophil-to-lymphocyte ratio at the start of treatment with OS or the outcome of the first re-staging. There are no statistically significant associations between baseline CRP levels or CRP kinetics in relation to tumor location or tumor stage.

**Figure 6:**
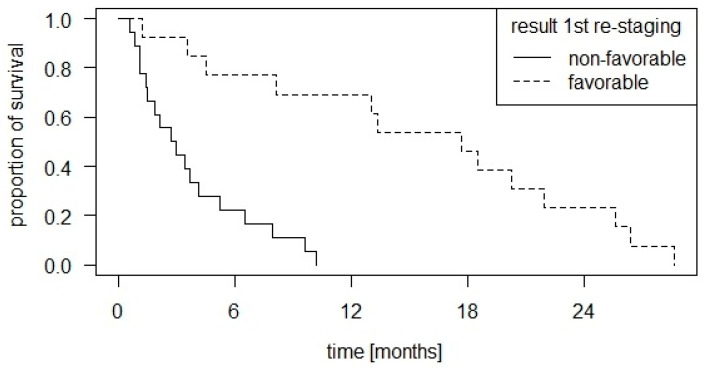
Kaplan–Meier curve of overall survival with group distribution according to the result of the first re-staging, showing a significant correlation between the result of the first re-staging after 12 weeks and OS in favor of favorable re-staging results (*p* < 0.001). Stable disease and remission were considered favorable, progression was considered non-favorable.

As expected, there is a significant correlation between the result of the first re-staging after 12 weeks and OS in favor of favorable re-staging results (*p* < 0.001). The survival curves are shown in Figure 6.

In a multivariate analysis using the Cox proportional hazards regression model, CRP kinetics according to our flare definition (*p* = 0.007) and the result of the first re-staging (*p* = 0.002) are the only independent prognostic factors for OS (Table 1). 

## 4. Discussion

CRP is a laboratory chemical marker that is routinely used in clinical work to detect inflammation and infection. Its determination is relatively inexpensive, and the measurement is well-established. It, therefore, seems suggestive to investigate its dynamics for the activation and activity of the immune system in general under CPI therapy.

The relationship between a patient’s individual inflammatory response and their prognosis in the context of HNSCC disease is repeatedly discussed. Various inflammatory response-related gene combinations have already been described, which have shown prognostic value in HNSCC patients [15,16]. The release of inflammatory proteins such as CRP can best be understood as activation of the immune system as part of the antitumor response. However, the mere increase or rise in CRP over time is not a good prognostic parameter (comparable to the non-responders in the definition, according to Fukuda et al.). This course would also be seen in the context of inflammatory complications of a growing tumor. In addition, it has already been shown that a chronic inflammatory state is associated with immunosuppression in the tumor microenvironment [17]. The most desirable course of CRP in the context of CPI therapy is, therefore, an initial rise (as a sign of activation of the immune system) followed by a subsequent fall (as a sign of functioning regulatory circuits and control of possible inflammatory complications), comparable to the flaring of a flame. 

In our study, baseline CRP alone showed no prognostic value with regard to overall survival. There was neither a correlation with the absolute values at the start of therapy nor a group difference between patients with a normal CRP at the start of therapy (≤5 mg/L) and patients with an elevated CRP. Further investigations, therefore, primarily looked at the dynamics/kinetic of CRP in the early phase of therapy.

With regard to the CRP group definitions of Ishihara et al., there were no significant differences in OS in our HNSCC patient collective. However, if we look at the corresponding Kaplan–Meier curve (Figure 4), we see a relatively steep decline in the “non-normalized” group in the early phase. Nevertheless, some patients from this group survive for a relatively long period of time, so they approach the other two groups at a later stage. It is possible that in a larger patient population, in which the survival of individual patients is less influential, a significant difference could still be found in favor of the “normal” and “normalized” groups.

The term “CRP flare” is not universally defined. In 2021, Fukuda et al. formulated the above definition for the groups “responder”, “non-responder” and “flare”. Based on this definition, there were no significant group differences in OS in our patient population and, accordingly, no prognostic value of CRP kinetic. In particular, the definition by Fukuda et al. that the CRP concentration must fall below the baseline after 3 months in the case of flare dynamics complicates the evaluation in our patient group, as 13 patients (29%) already had normal CRP levels at the start of treatment. These patients were, therefore, already excluded from the definition of a flare responder or a responder. 

In 2023, a group of researchers compared the CRP group definitions of Fukuda and Ishihara in a larger number of patients with renal cell carcinoma [18]. They found a superior progression-free survival (PFS) in patients who were assigned to CRP-flare and CRP-responders, according to Fukuda et al., but no significant differences in OS. With regard to Ishihara et al.’s definition, both a longer PFS and a longer OS were found in patients in the normal and normalized group. At least for the renal cell carcinoma entity for which the earlier CRP group definition was established, CRP kinetic, therefore, appears to have prognostic significance. However, the authors point out that the timing and frequency of CRP measurements still need to be optimized.

In the two previously postulated definitions by Fukuda and Ishihara, fixed time periods are proposed for the classification of CRP kinetic. In our definition, we propose an evaluation of the first three measurements (baseline concentration at the start of CPI therapy and two further control measurements before each of the following administrations). This takes into account the different pharmacokinetics of the two CPI drugs established in the HNSCC area, nivolumab (2-weekly administration) and pembrolizumab (3-weekly administration). This may also be a reason why the conventional CRP definitions did not allow any prognostic significance in our patient population.

It must be emphasized that in most previous studies on CRP dynamics in other entities, only patients undergoing nivolumab therapy were examined. So far, the only other study on HNSCC patients has exclusively examined pembrolizumab patients [12], but this also included patients with concurrent chemotherapy. In our cohort, both nivolumab and pembrolizumab patients were studied, as this represents the clinical reality for R/M-HNSCC patients. The decision as to which of the two drugs a patient receives was based on the Combined Positive Score (CPS, proportion of PD-L1-positive tumor and immune cells in relation to all tumor cells, multiplied by 100), with patients with a CPS ≥ 1 receiving pembrolizumab and those with a CPS < 1 receiving nivolumab. While almost twice as many patients received pembrolizumab than nivolumab, the baseline CRP concentration tended to be higher in the nivolumab group (42 vs. 22 mg/L), but the difference failed to reach the statistical significance level (*p* = 0.08). 

R/M-HNSCC patients in whom CPI therapy is initiated represent a heterogeneous patient population. It includes patients in relatively good general condition with non-operable metastasis as well as severely ill patients with superinfected, skin-breaking primary tumors or metastases. This alone explains the relatively large standard deviation in the baseline CRP concentration in our collective.

It could be assumed that in primary “sterile” tumor localizations (e.g., renal cell carcinoma), the tumor may be less susceptible to infection. However, at least the baseline CRP levels were comparable (23 mg/L in renal cell carcinoma [7] and 17 mg/L in our HNSCC collective). Nevertheless, the baseline CRP level in the HNSCC collective showed a significantly wider spread than in the renal cell carcinoma collective (0–118 mg/L vs. 0–64 mg/L). 

Despite this, several cofounders can be assumed not only with regard to the baseline values but also to CRP kinetic under CPI therapy in HNSCC patients.

Chronic consumption of noxious substances itself also leads to a permanent inflammatory state in the patient’s body and can cause changes in CRP [19]. In our patient population, 30 patients (68%) consumed nicotine. However, there were no significant differences in baseline CRP levels between smokers and non-smokers (*p* = 0.22). Furthermore, there were no significant associations between smoking behavior and CRP group assignment (by Fukuda’s definition: *p* = 0.35; by our own definition: *p* = 0.85). Thus, it could be assumed that nicotine consumption had no significant influence on the assessment of CRP dynamics under CPI therapy, at least in our collective. 

There were also no significant differences in the baseline CRP levels with regard to gender, p16 status and different age groups.

Furthermore, typical complications in HNSCC patients, such as aspiration pneumonia, can falsify the evaluation of CRP as interim acute inflammatory phases as well. Retrospectively, it is difficult to determine whether CRP dynamics were due to a response to CPI therapy or to an infection or inflammation. It can only be stated that in the case of a manifest infection, CPI therapy would have been paused in clinical practice.

Immunosuppressive drugs such as steroids can also have an impact on CRP kinetic. For example, Klumpner et al. described a lack of prognostic benefit of CRP kinetic in a subgroup of patients who also received steroid therapy during CPI therapy [20]. Because no patients in our HNSCC collective were administered steroids in parallel with CPI therapy, we cannot draw any conclusions in this regard.

As expected, there was a significant association between a favorable first re-staging and OS. If patients do not respond to the initiated immunotherapy, tumor progression and death can occur rapidly. Indirectly, it can thus be concluded whether a relevant proportion of tumor cells escape immunosurveillance via the PD-1 pathway, as CPI therapy with nivolumab or pembrolizumab would then be more likely to mediate a treatment response [21]. Nevertheless, it is possible that other immunoescaping mechanisms of the tumor cells may gain the upper hand in the further course of therapy, resulting in resistance to CPI therapy. Progressive disease would then be possible again at a later date. However, these patients then have a longer OS than patients who are already resistant to CPI therapy at the start of treatment.

With regard to the first re-staging, there was a significant correlation with the CRP group definitions according to Ishihara (*p* = 0.028), whereby the “normal” and “normalized” were associated with a favorable re-staging and the “non-normalized” with a non-favorable result. For the CRP group definitions, according to Fukuda or our definition, no associations were found with the result of the first re-staging. Thus, a distinction must be made between the prognostic value in relation to OS and the predictive value in relation to the specific treatment response.

As discussed above, the conventional definition of flare dynamics, as examined in several studies on various tumor entities, does not appear to be suitable for patient collectives that have normal CRP levels at the start of therapy. Using a simplified version of this definition, however, we were able to demonstrate the apparent prognostic significance of early CRP dynamics under CPI therapy in such an HNSCC collective. 

## 5. Limitations

The limitations of this study are the size of the patient population due to the relatively new form of therapy and its retrospective nature. Individual reasons for fluctuations in CRP (such as acute infections) cannot be reliably identified retrospectively. Another limitation is the high heterogeneity of the patient population. Because we included patients in different lines of therapy, we inevitably had to compare therapy-naïve and previously treated tumors with correspondingly different biology. The general condition of the patients was also very heterogeneous, and the overall survival must be considered with corresponding caution, as non-tumor-associated deaths cannot be ruled out.

## 6. Conclusions

The early dynamics of CRP do indeed appear to have a certain prognostic value in HNSCC patients undergoing CPI therapy. As a cheap and readily available parameter, its use seems promising. However, interpretation is difficult due to multiple confounding factors and must be undertaken with caution. Studies on larger patient populations, as well as studies on the underlying pathophysiological mechanisms, could further improve the usability of CRP as a prognostic parameter in CPI therapy of R/M-HNSCC patients.

## Figures and Tables

**Table 1:** Univariate and multivariate analysis for overall survival. OS: overall survival. HR: hazard ratio. CI: confidence interval. CRP: C-reactive protein. Ref.: reference.

		Univariate		Multivariate		
Variables		*p*-Value	Median OS [Days]	*p*-Value	HR	95% CI
Age [years]	≤70	0.8	146			
	>70		124			
Sex	Male	0.5	238			
	Female		115			
p16	Positive	0.6	238			
	Negative		157			
Result 1st re-staging	Favorable	<0.01	529	<0.01	0.06	0.014–0.222
	Non-favorable		84.5	Ref.	Ref.	Ref.
CRP group by Fukuda et al. [7]	Non-responder	0.85	124	0.20		
	Responder	0.80	216	0.18		
	Flare	Ref.	308	Ref.		
CRP group by Ishihara et al. [13]	Normal	0.46	266	0.89		
	Normalized	0.08	238	0.98		
	Non-normalized	Ref.	95	Ref.		
CRP group by own definition	Non-flare	0.05	102	<0.01	4.3	1.55–11.9
	Flare		297	Ref.	Ref.	Ref.

## Data Availability

The data presented in this study are available in this article (and Supplementary Material Table S1).

## Supplementary Materials

The following supporting information can be downloaded at: https://www.mdpi.com/article/10.3390/cancers16132424/s1, Table S1: The data presented in this study are available in this article.
